# Wound Coverage, Adjuvant Treatments, and Surgical Outcomes for Major Keloid Scars: A Systematic Review and Meta-Analysis

**DOI:** 10.1093/asjof/ojae129

**Published:** 2024-12-26

**Authors:** David Cardenas, Trey Cinclair, Anca Dogaroiu, Chandler Hinson, Matthew Sink, Brandon Bruce, Andrei Odobescu, Douglas Sammer, Andrew Y Zhang

## Abstract

Patients suffering from keloid scarring often face debilitating functional and psychosocial symptoms. The complex pathophysiology of keloids necessitates a patient-centered therapeutic regimen to optimize patient satisfaction and disease resolution. This is especially challenging for patients with major keloid scars that once surgically resected, leave a defect that cannot be closed primarily. The authors intended to identify existing therapeutic regimens reported for the treatment of major keloids and detail the clinical and patient-satisfaction outcomes for those approaches. A systematic review and meta-analysis was designed in accordance with Preferred Reporting Items for Systematic reviews and Meta-analyses guidelines, querying 2 databases along with a manual search for relevant literature. This review identified 10 studies, totaling 244 patents. All patients underwent surgical resection of their keloid. Subsequent therapeutic regimens included a variety of coverage techniques, including skin grafts, perforator flaps, closure with secondary intention, and skin substitute placement. Additionally, there were multiple adjuvant therapies utilized such as radiotherapy and steroids. The overall keloid recurrence rate was 21%. Patients who received wound coverage had a lower rate of keloid recurrence compared with those treated by secondary intention. When available, patients receiving local flap coverage plus adjuvant radiotherapy were found to have significantly lower keloid recurrence rates and higher patient satisfaction. Although these findings support surgical excision and radiation therapy as the mainstay treatment for major keloids, the efficacy of various wound coverage options, especially when local perforator flaps are not available, requires continued investigation. A gap exists on guidelines for postoperative adjuvant therapies.

**Level of Evidence: 3 (Therapeutic):**

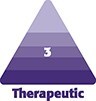

Keloids are thick, raised scars that result from abnormal wound healing in response to skin trauma or inflammation.^1^ Keloids manifest as excessive, dense fibrous tissue that extends beyond the original wound's borders.^2^ These lesions commonly present clinically as raised, firm, and irregularly shaped papules or plaques of various colors that do not regress spontaneously.^3^ Keloids are often symptomatic, with 80% of patients reporting pain, itching, or a burning sensation.^4,5^ There are multiple factors that increase an individual's risk for developing keloids. Studies have shown that keloid formation has a strong genetic susceptibility in people of African and Asian descent, with incidence rates reported as high as 16% in these populations.^6,7^ However, there has been no specific gene identified that directly correlates with the development of keloids.

Although the understanding of keloid pathophysiology and epidemiology has improved over the years, the pathogenesis for keloids is not completely understood.^8^ Treatment modalities vary widely from institution to institution based on anecdotal experiences and studies with differing patient population, disease site, treatment protocols, and length of follow-up.^3,9-11^ Smaller keloids are often treated on an outpatient basis in a dermatologist's office with nonsurgical modalities.^3^ However, large keloids often require surgical intervention in combination with adjunctive therapy.^12^

The most difficult challenge in treating keloids is in preventing recurrence.^3^ Surgical excision as a monotherapy has reported recurrence rates from 45% to 100%.^13-16^ Treatment is even more challenging for “major keloids,” which the authors define as keloids that cannot be closed primarily after excision. Despite over a century of efforts, recurrence remains high and are the most difficult and unpredictable aspect of major keloid treatment.^3^ For keloids undergoing surgical excision, multiple studies have shown one of the key technical tenets to reduce recurrence is tension-free primary closure.^17-21^ However, surgeons can be faced with massive keloid lesions that will result in large open wounds that cannot be closed after excision. Surgeons face significant challenges when treating major keloids because of the complex nature of these cases. Patients with major keloids stand to benefit the most from effective treatment, yet also present the highest risk of recurrence because of the large wounds that cannot be closed primarily with a tension-free technique. As a result, surgeons must carefully consider the best combination of surgical techniques and adjuvant therapies to optimize outcomes while minimizing the risk of keloid reformation.

Although keloids are often symptomatic and functionally limiting, they may also cause significant psychosocial distress because of their undesirable appearance.^22^ Additionally, keloid patients score significantly higher on surveys assessing anxiety, interpersonal relationship sensitivity, and depression when compared with patients with no keloid scars.^22^ Patients with major keloids are the most desperate for treatment because of the significant negative social psychological impact of these disfiguring and symptomatic lesions.^22,23^

There is much room for improvement in treatment approaches of keloids. A unifying treatment algorithm for major keloid, let alone keloids in general, is monumentally challenging. Keloids behave differently in different patient populations and locations. Treatment modalities vary greatly in existing literature. Our systematic review and meta-analysis aims to outline these differences and control known variables to identify beneficial factors for surgical management of major keloids.

## METHODS

### Strategy and Registration

This systematic review and meta-analysis performed in accordance with the Preferred Reporting Items for Systematic reviews and Meta-analyses (PRISMA) guidelines.^24^ This systematic review was registered on PROSPERO with details of the initial protocol under the following ID: CRD42024555153.

### Search and Data Sources

A literature search was performed using Embase, MEDLINE, and a manual search. Articles were extracted from these databases on May 2, 2024. In order to find as many relevant articles, all articles available at the date of the search were considered. Predetermined search terms were used in these databases (Appendix).

### Selection Criteria

Articles obtained were imported into the Covidence platform (Veritas Health Innovation) for screening. Screening was independently performed by 2 investigators (D.C. and T.C.) by title and abstract, and later by full text review. Conflicts were resolved by a third reviewer (A.Y.Z.). The predefined study population (*P*), intervention (*I*) or exposure (*E*), comparison (*C*), outcome parameters (*O*), and study type (*S*) (*PI*(*E*)*COS* factors) for eligibility of the studies are: *P* = adults and children 15 and older with major keloids as defined by inability to be closed primarily after excision; *I* = surgical excision of keloids with adjuvant therapies; *C* = surgical excision without adjuvant therapies; *O* = recurrence rates for major keloids following treatment (primary); *O* = patient satisfaction with keloid treatment (secondary); and *S* = randomized and nonrandomized control trials, case control or cohort studies, case series.

To standardize definitions, recurrence is the presentation or reappearance of a keloid scar in part or in the entirety of the original surgical site. Reappearance of the keloid scar had to be diagnosed by a member of the medical team and not patient reported.

The exclusion criteria were as follows: studies not published in English, reviews, meta-analyses, guidelines, case reports, case series with <5 patients, and studies not relevant to “major” keloid treatment.

### Data Extraction

Using a data extraction form, the following variables were extracted from the included studies: patient demographics (age, ethnicity), keloid scar surface area, location of keloid scar, skin closure technique (perforator flap, skin graft, skin substitute, and secondary intention), radiotherapy, adjuvant treatments, recurrence rate, patient satisfaction, and clinical follow-up period.

### Statistical Analysis

A meta-analysis was performed to compare rates of recurrence among different methods of closure along with overall patient satisfaction. Chi-squared analysis was conducted within Microsoft Excel 2022 (Microsoft Corporation, Redmond, WA) to determine statistical significance between treatment cohorts regarding recurrence of keloids and patient-reported satisfaction with keloid treatment.

### Risk of Bias Assessment

A quality and risk of bias assessment was performed based on the Newcastle–Ottawa Scale (NOS).^25^ All studies were also assigned a level of evidence rating based on the American Society of Plastic Surgeons Evidence Rating Scale for Therapeutic Studies, organized into classes (I, II, III, IV, and V).^26^

## RESULTS

**Figure 1.**
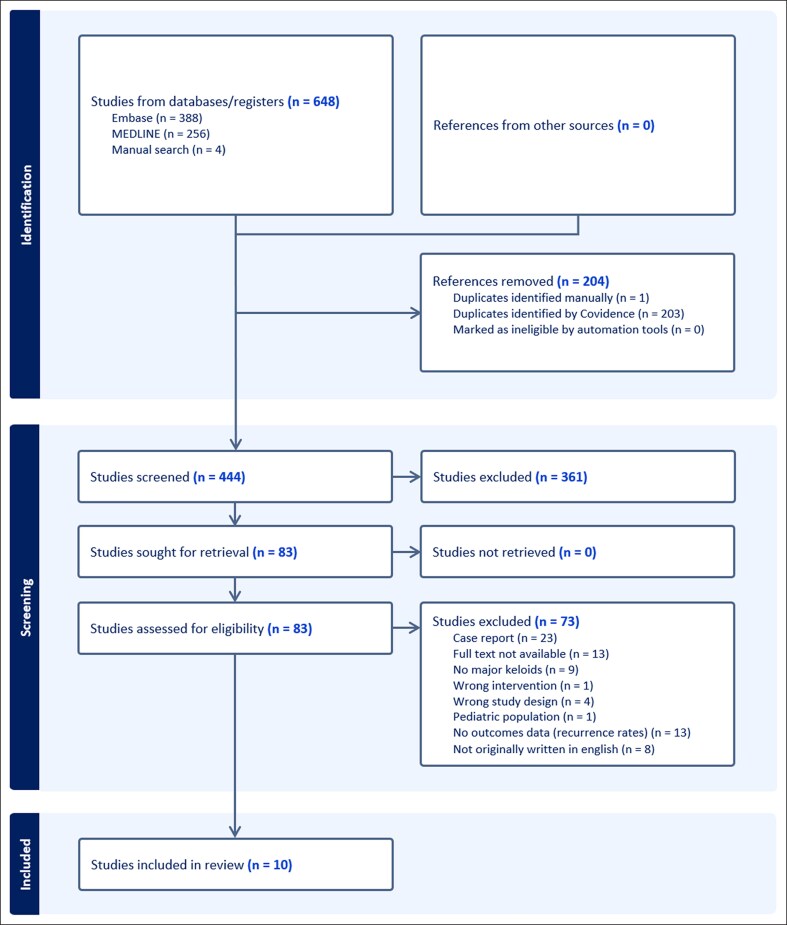
Flow diagram of search and study selection on the basis of the Preferred Reporting Items for Systematic reviews and Meta-analyses guidelines.

The initial search yielded 648 articles. After removing duplicated studies and removing those that did not meet the inclusion criteria, 10 remained (Figure 1). The characteristics and data of the included studies are shown in Table 1.

**Table 1.** List of Studies Included in the Systematic Review and Meta-analysis

Author, year	Study design	Sample size, *n* (m:f)	Age, mean (SD), (range)	Ethnicity (%)	Keloid scar surface area, mean (SD) (range)	Keloid location (%)	Surgical intervention(s)	Adjuvant treatment (frequency)	Radiation treatment (frequency)	Follow-up period, mean (SD)	Keloid recurrence rates	NOS score	LOE
Boccara et al, 2021	Retrospective Case Series	42 (31:11)	37.7 years (12.1), (27.5-41.5)	Not reported	23.5 cm^2^ (29.7), (3.5-32 cm^2^)	Torso = 16 (28%) Cervicofacial = 15 (26.3%) Scalp = 7 (12.3%) Occipital scalp = 6 (10.5%) Limbs = 7 (12.3%) Pubis = 7 (12.3%) Auricle = 5 (8.8%)	Group 1: healing by secondary intention with adherent postoperative steroids Group 2: healing by secondary intention with nonadherent postoperative steroids	Group 1: adherent postoperative steroids Group 2: nonadherent postoperative steroids	None	14.1 months (8.1)	Group 1: 60% Group 2: 81%	7	IV
Stucker et al, 1992	Retrospective Case Series	37 (24:13)	Not reported	Black (100%)	Not reported	Face and neck = 27 (75%) Neck and chest = 9 (25%)	CO_2_ laser excision	Postoperative steroids and hyaluronidase every 3 weeks for 6 months	None	36 months	19%	6	IV
Liu et al, 2021	Retrospective Case Series	15	40 years (22-63)	Asian (100%)	(48-315 cm^2^)	Chest = 15 (100%)	Surgical excision with expanded parasternal intercostal perforator flap coverage	None	2 sessions, 9 Gy per session, POD 1 and POD 8	Not reported	0%	8	IV
Song et al, 2018	Retrospective Case Series	35	45.3 years (24-67)	Asian (100%)	48 cm^2^ (30-70 cm^2^)	Chest = 35 (100%)	Surgical excision with intercostal perforator flap coverage	Postoperative topical antiscarring drugs for 6 months^a^ and 6 months of continuous pressure therapy	2 sessions, 9 or 10 Gy per session, POD 1 and POD 8	24 months	0%	7	IV
Ogawa et al, 2016^27^	Retrospective Case Series	10 (9:1)	37.9 years (22-55)	Asian (100%)	Minimum area = 40 cm^2^	Chest = 10 (100%)	Surgical excision with internal mammary artery perforator propeller flap coverage	Postoperative Silicone-tape fixation for 6 months	4 sessions, 5 Gy per session, POD 5-8	28.7 months (range, 18-42)	0%	8	IV
Zhao et al, 2023	Retrospective Case Series	25 (8:17)	63 years (24-79)	Asian (100%)	43.57 cm^2^ (12.29)	Chest = 25 (100%)	Surgical excision with internal mammary artery perforator propeller flaps	Postoperative silicone gel sheets for 6 months	5 sessions, 5 Gy per session, POD 1-5	18 months (range, 12-32)	0%	9	IV
Li et al, 2014^28^	RCT	Group 1: 29 (19:10) Group 2: 24 (13:11)	Group 1: 23 years (5) Group 2: 21 years (6)	Asian (100%)	Group 1: 52.08 cm^2^ Group 2: 53.56 cm^2^	Chest = 53 (100%)	Group 1: surgical excision with skin graft coverage Group 2: presurgical radiotherapy followed by surgical excision with skin graft coverage	None	Group 1: 2 sessions, 9 Gy per session, POD 10 and POD 17 Group 2: 2 sessions, 9 Gy per session, preoperative Day 1 and POD 14	12 months	Group 1: 55% Group 2: 17%	8	III
Wang et al, 2015	Prospective Case Series	Group 1: 9 (5:4) Group 2: 6 (4:2)	Group 1: 45.33 years (8.94) Group 2: 46 years (5.76)	Asian (100%)	Group 1: 60.16 cm^2^ (10.95) Group 2: 75.08 cm^2^ (17.22)	Chest = 10 (66.7%) Abdominal = 5 (33.3%)	Group 1: surgical excision with internal mammary artery perforator propeller flaps Group 2: surgical excision with superior epigastric artery perforator propeller flaps	None	Group 1: 5 sessions, 3.5 Gy per session, POD 3-7 Group 2: 4 sessions, 4 Gy per session, POD 3-6	23.4 months	Group 1: 0% Group 2: 0%	8	IV
Dong et al, 2024	Retrospective Case Series	Group 1^b^: 14 (6:8) Group 2: 5 (3:2) Group 3: 2 (1:1)	Group 1^b^: 41.13 years (18.9) Group 2: 38.6 years (16.41) Group 3: 54.5 years (20.51)	Asian (100%)	Group 1^b^: 24.82 cm^2^ (15.36) Group 2: 17.8 cm^2^ (7.56) Group 3: 41.5 cm^2^ (9.19)	Chest = 10 (47.6%) Abdomen = 3 (14.3%) Suprapubic = 3 (14.3%) Other = 7 (23.8%)	Group 1: surgical excision z-plasty closure^b^ Group 2: surgical excision with skin graft coverage Group 3: surgical excision with abdomen or right groin perforator flap	None	Groups 1-3: 3 sessions, 6 Gy per session, POD 1-3	Group 1: 78.64 months (43.73) ^b^ Group 2: 80.6 months (20.17) Group 3: 37 months (24.04)	Group 1: 14.29%^b^ Group 2: 0% Group 3: 0%	7	IV
Davison et al, 2020	Prospective Case Series	5 (0:5)	45.2 years (18.62)	African American (80%) Hispanic (20%)	52.6 cm^2^ (45.15)	Chest = 5 (100%)	Surgical excision with integra (1-5)	Silicone gel (1) Intraoperative injection of 50:50 5-U and triamcinolone and silicone sheeting (1) Intraoperative injection of triamcinolone and silicone sheeting (1) Intraoperative injection of 80:20 5-FU and triamcinolone and silicone sheeting (1) Intraoperative injection of 5-FU and silicone sheeting (1)	1 session, POD 0, 800 cGy (3) 1 session, POD 17, 800 cGy (2.)	45.4 months (20.89)	0%	8	IV

Newcastle–Ottawa scale (NOS) score and level of evidence (LOE) based on the American Society of Plastic Surgeons evidence rating scale for therapeutic studies. SD, standard deviation. ^a^Topical antiscarring drugs not specified in the article. ^b^The patient cohort from this study who underwent *z*-plasties for closure were excluded from meta-analysis.

### Demographics

A total of 244 patients were included in this study. The patients had a mean age of 39.2 years (range, 22-79 years) and mean keloid surface area of 50.94 cm^2^ (range, 3.5-315 cm^2^). A total of 161 patients (80%) were Asian and 41 (20%) were African American. The most common location of major keloids included chest (59%), face and neck (15%), and abdomen (3%). Of note, Stucker et al (*n* = 37) was not included in mean age as the study did not report ages among their study population. Additionally, Boccara et al (*n* = 42) was not included in ethnicity as the study did not report the ethnicity of their patient population.

### Risk of Bias and Quality Score

The risk of bias and quality assessment for the included studies were conducted using the NOS. The average NOS was 7.6 (range, 6-9; Table 1). All studies were assigned a level of evidence of IV except for Li et al which received a score of III.

### Outcomes

**Figure 2.**
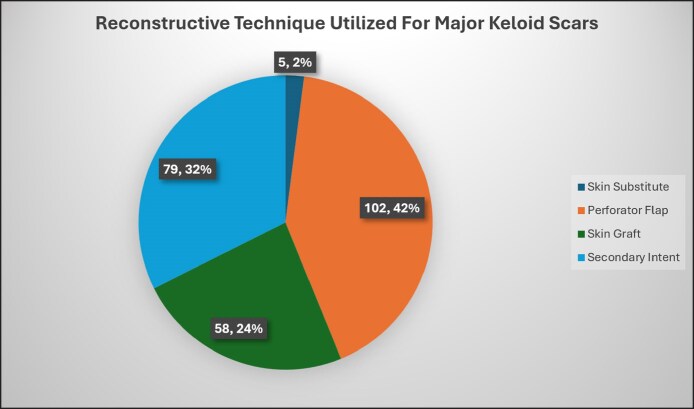
Pie chart representing the techniques utilized for reconstruction of major keloid scars from studies included in the systematic review and meta-analysis.

The primary outcome of this study was recurrence of keloids after treatment for major keloids. Of the total 244 patents included in this study, the recurrence rate was 21% (*n* = 51). When stratified by closure technique, there were 4 different approaches utilized in this patient cohort: closure by secondary intention (79, 32%), split thickness skin graft (58, 24%), skin substitute (5, 2%), and perforator flap (102, 42%; Figure 2). The majority of this patient cohort received either preoperative, intraoperative, or postoperative radiotherapy (165, 68%). In addition to radiotherapy, a subset of patients (79, 32%) received further adjuvant therapy including glucocorticoids (topical or injection), topical fluorouracil (FU), and/or continuous pressure wound therapy. Those who received surgical excision with radiotherapy and adjuvant therapy had a lower rate of recurrence (0%) when compared with surgical excision and radiotherapy alone (16%; *χ*^2^ = 7.04, *P* = .01).

### Radiation

Of the total patient sample, 165 (68%) patients received radiotherapy in addition to surgical excision of their keloids. Most patients (141, 58%) received radiotherapy after surgical excision and wound coverage, whereas a smaller cohort received radiotherapy before surgical treatment (24, 10%). All patients that underwent coverage with a perforator flap, skin graft, or skin substitute received radiotherapy, with the only group not receiving radiotherapy being those treated with secondary intention. Since radiotherapy was not utilized in the secondary intention treatment group, we were unable to compare the impact of radiotherapy between wounds treated with coverage (skin grafts, flaps, or skin substitute) and those managed by secondary intention.

### Type of Coverage

When stratified by type of closure, recurrence was statistically significant based on technique (*χ*^2^ = 50.815, *P* < .01). Table 2 stratifies rates of recurrence by type of wound coverage. Excluding secondary intention (because of not receiving radiotherapy) and skin substitute (because of small sample size), perforator flaps had a statistically lower rate of recurrence (0%) when compared with skin grafts (34%) when controlling for radiotherapy (*χ*^2^ = 53.72, *P* < .01).

**Table 2.** Rates of Keloid Recurrence Stratified by Technique of Skin Closure

	Perforator flap, *n* (%)	Skin graft, *n* (%)	Secondary intention, *n* (%)	Dermal substitute, *n* (%)
No. of patients with recurrence	0 (0)	20 (34.48)	31 (39.24)	0 (0)
No. of patients without recurrence	102 (100)	38 (65.52)	48 (60.76)	5 (100)
Total	102	58	79	5

### Adjuvant Medical Therapy

Postoperative adjuvant therapies of the studies included steroid injections (83, 34%), hyaluronidase injections (37, 15%), silicone gel sheets (40, 17%), continuous pressure therapy (35, 14%), 5-FU (2, 1%), and nonspecified topical antiscarring drugs (35, 14%). Intraoperative adjuvants included the use of steroid injections and 5-FU. The frequency of use for postoperative steroid and hyaluronidase injections varied widely in the studies, ranging from every 2 weeks to every 3 months.

### Patient Satisfaction

Five studies (50%) reported patient satisfaction after treatment of their keloids. The treatments assessed for satisfaction included surgical excision with perforator flap and radiotherapy (*n* = 65) and surgical excision with skin graft and radiotherapy (*n* = 24). Mean follow-up time for assessing patient satisfaction in this cohort was 16.38 months. Patients who received surgical excision with perforator flap and radiotherapy had a statistically higher level of satisfaction (100%) compared with those who received surgical excision with skin graft and radiotherapy (92%; *χ*^2^ = 5.54, *P* = .02).

## DISCUSSION

In this study, the authors conducted a systematic review and meta-analysis detailing the reported outcomes of various treatment protocols for major keloids in the existing literature. The data isolated from the existing literature identified through this review suggest that for major keloids, surgical excision with radiation, with or without other adjuvant therapies, results in the best clinical outcomes. This systematic review demonstrates the limited number of studies that specifically investigate major keloids, the available treatments modalities, and their associated outcomes. The authors share these findings to demonstrate the described treatment options and outcomes for major keloids.

This study found that the lowest recurrence rates were reported when surgical excision was followed by wound coverage with postoperative radiation therapy. In contrast to wound coverage, the highest recurrence rates were observed in studies where healing was left to secondary intention without the use of radiation. Satisfaction rates were higher in patients who received perforator flaps with radiation treatment when compared with skin grafting with radiation. Not surprisingly, the recurrence rates for flaps with radiation were also significantly lower when compared with skin grafting with radiation. Notably, only 2 of the studies reviewed did not employ postoperative radiation and had higher relative recurrence rates, supporting the common clinical practice of using radiation in conjugation to prevent recurrence of major keloids. However, the impact of radiotherapy could not be assessed in this study because the majority of patients included in this study received radiotherapy. Additionally, the only patients who did not receive radiotherapy also lacked wound coverage through a perforator flap, skin graft, or skin substitute. This created the statistical inability to separate the effects of radiotherapy from those who received wound coverage through a perforator flap, skin graft, or skin substitute.

The studies in our review that incorporated radiotherapy had significant variation in the number and timing of radiotherapy doses; yet cohorts that received any radiation had a lower risk of recurrence. Radiation as an adjuvant therapy to surgical excision has been reported to be used as early as 1906.^29^ However, it was not until the 1940s and 1950s that postoperative radiation was recommended in the treatment of keloids.^30,31^ Combining surgical excision with radiation therapy has been reported to significantly lower the recurrence rate compared with surgery alone (23% with radiation added vs 45%-100% with surgery alone).^3^ Advancements of electron beam radiation and brachytherapy has been reported to reduce recurrence rates for keloids to as low as 12%.^32,33^ Numerous studies and reviews have explored different radiotherapy techniques, including the appropriate radiation dose, dose–response relationship, and fractionation schemes. Techniques, such as kilo-electron volt X-ray, mega-electron volt electron beams, and brachytherapy, have been utilized in treating keloids. However, a universally accepted protocol for selecting the type of radiotherapy, dose, and fractionation for treatment has yet to be established.^34-36^ Among the included studies in this review, radiation was often employed postoperatively with 1 to 5 sessions with Gray radiation dosages between 5 and 10. Early postoperative adjuvant radiation therapy has also been proposed to enhance success rates in keloid treatment.^37-39^ Despite improvements in recurrence rates, the reported rates vary considerably, ranging from 12% to 70%.^14,39-41^ This inconsistency is often because of the heterogeneity in scar locations and etiologies, patient demographics, and variations in radiation protocols employed across studies. Eight of the studies in our review incorporated radiotherapy as an adjuvant, totaling for 165 patients with a combined recurrence rate of only 13%. These data fall on the lower end of reported recurrence for all keloids with postoperative radiotherapy.

There is evidence that radiotherapy as an adjuvant treatment for major keloids provides improved outcomes. A growing body of well-designed studies has also confirmed the comparative effectiveness of combining surgical excision with postoperative irradiation therapy.^42^ A consecutive study involving 78 keloids from 40 patients showed that immediate postoperative radiotherapy reduced the recurrence rate compared with surgical excision alone.^43^ Long-term retrospective studies have further highlighted the importance of postoperative irradiation in keloid management. For instance, Kovalic and Perez followed 75 patients with 113 keloids over an average period of 9.75 years, reporting an overall recurrence rate of 27%.^44^ Numerous other studies have largely supported the efficacy of irradiation as an adjuvant therapy. One study comprised mostly of ear keloids noted a total recurrence rate of 2% in 250 patients over 50 years.^45^ These studies support why radiotherapy is so often used for major keloids, despite none of these studies looking exclusively at major keloids. However, it is important to note that smaller keloids can respond vastly different to treatment when compared with major keloids, and data in these studies cannot be consistently or accurately applied to major keloids.^46^

**Figure 3.**
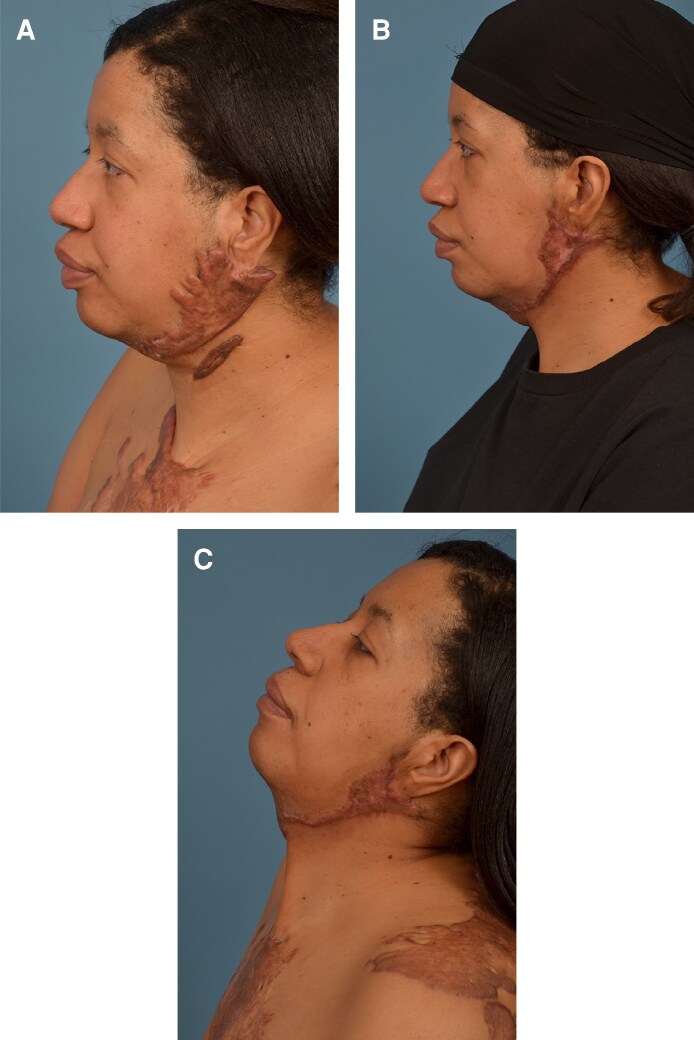
(A) A 42-year-old female patient presented with extensive recurrent keloids to the left and right jawlines. Patient reported symptoms of intermittent drainage and tenderness of keloids. Notably, a prominent aspect of her presentation pertained to the pronounced emotional distress arising from the keloids, manifesting as feelings of embarrassment within social contexts. (B) Patient 6 months postoperatively after excision and use of Integra for wound coverage, without a skin graft. (C) Patient shown at 1 year postoperatively with no signs of recurrence. Patient expressed her happiness and satisfaction with the results.

To better understand the effects of wound coverage and keloid recurrence, this study directly compared perforator flaps, skin graft, and skin substitute such as Integra (Integra LifeSciences, Princeton, NJ). Our review showed perforator flaps with immediate postoperative radiation reduced recurrence rates to a statistically significant degree when compared with skin grafts with delayed radiotherapy. This finding of higher recurrence rates for skin grafts for major keloids agrees with other data in the literature for nonmajor keloids.^46^ Skin substitutes, like Integra, showed no recurrence; however, the sample size in this cohort was small. The authors of this article have experience utilizing skin substitutes for wound coverage after keloid resection, which has demonstrated satisfactory patient-reported outcomes along with minimal keloid recurrence (Figure 3). Radiotherapy is most effective when administered on the day of surgical excision and no later than 48 h postoperatively.^47^ However, in patients undergoing skin graft procedures, radiotherapy within this timeframe is challenging because of the presence of surgical dressings and concerns regarding graft viability. Consequently, radiotherapy treatment is sometimes deferred until ∼10 to 14 days after surgery, by which time graft survival is generally assured. It is hypothesized this delay in radiotherapy leads to increase recurrence rates. Li et al showed that early stage radiotherapy, achieved by radiotherapy treatment a day before excision of major keloids, resulted in a decrease in recurrence (16%) when compared with delayed radiotherapy (55%) of skin grafting.^28^ The data in this meta-analysis supports this, as the recurrence rates for skin grafts were higher when compared with flaps and skin substitutes. Skin flaps are highly vascular and less prone to vascular damage from radiotherapy as skin grafts. Ogawa et al showed the use of internal mammary artery perforator pedicled flaps combined with immediate postoperative radiotherapy in patients with major keloids leads to no complications or recurrences during a mean follow-up period of 28 months.^27^ However, there is a paucity of studies, particularly prospective randomized controlled trials with large sample sizes, that have assessed the complications of postoperative radiotherapy between skin grafts and perforator flaps. Local flap coverage after keloid excision is limited to small- and medium-sized lesions on the chest, limiting its applicability to other regions prone to major keloids such as the head and neck. Additionally, there is evidence supporting that increased time intervals between surgery and radiotherapy negatively correlates with clinical outcomes.^48,49^ Seegenschmiedt et al and Wagner et al showed lower recurrence rates with smaller postoperative radiotherapy intervals.^50,51^

Skin substitutes, such as Integra, are an emerging treatment option that has shown positive outcomes in regards to minimizing recurrence and symptoms.^12,52-54^ Our review only included 1 article with 5 patients who underwent wound coverage with a skin substitute with results showing no recurrence. Though only a small sample size, the use of skin substitute for wound coverage showed comparable results to perforator flaps. A benefit of using a skin substitute is it removes the risk of additional scarring or keloids from the donor sites used by flaps and skin grafts.^12,52^ Additionally, skin substitutes can be utilized when local perforator flaps are not available for wound coverage. Although case reports were not included in this meta-analysis, there are multiple case reports detailing the use of a skin substitute for keloids and none reported recurrence.^12,52-55^ Although case reports are subject to bias and limited by low sample sizes, skin substitutes provide a promising option for treatment of major keloids. Nguyen et al present a case series of 5 patients who underwent surgical excision of keloids with bilaminar skin substitute and epidermal skin grafting placement showing no recurrence.^52^ Interestingly, 3 of these patients did not receive postoperative radiotherapy yet there was no reported recurrence. The hypothesis for the utility of a skin substitute is promoting ordered dermal regeneration and providing a moist and sterile wound healing favorable environment.^56^ Other hypotheses include altering the wound environment from inflammatory to regenerative, minimizing the amount of inflammatory cells often seen with flaps and skin grafts. Additionally, skin substitutes can decrease the tension on the wound.

Our findings suggest that although the combination of surgical excision, wound coverage, and radiation remains a cornerstone in the treatment of major keloids, the existing evidence is insufficient to determine the most optimal approach. The limited number of studies that met our inclusion criteria underscores a significant gap in the literature, indicating a need for more comprehensive research that focuses on large keloids that leave large soft tissue defects. Future studies should aim to explore alternative methods, such as the use of skin substitutes, to help in improving the standard treatment regimens for keloids. This research would involve investigating different types of coverage methods, optimizing radiotherapy and adjuvant therapies, and potentially integrating new therapies.

### Limitations

There are multiple limitations to this study. With regards to radiotherapy, the patient cohort that did not receive radiotherapy directly overlapped with the patient cohort treated by secondary intent, making it difficult to determine whether the higher rate of keloid recurrence was because of lack of radiotherapy or differences in wound coverage. Additionally, there was no standardized treatment protocol for radiotherapy, with some patients receiving a greater quantity of adjuvant radiotherapy. This study was unable to assess the effect of timing and quantity of radiotherapy treatments on keloid recurrence. However, future studies should directly study the correlation between quantity and intensity of radiotherapy treatment to keloid prevention.

## CONCLUSIONS

Although surgical excision combined with radiation therapy continues to exhibit being the mainstay treatment for major keloids, there is a clear and pressing need for further studies. Current treatment methods, though effective to some extent, often fall short of providing lasting relief. Our analysis highlighted that the lowest recurrence rates occurred when surgical excision was followed by wound coverage using perforator flaps or skin substitutes with postoperative radiation therapy. Emerging treatments, like skin substitutes (eg, Integra), show promise but require further validation because of limited evidence. The review highlights the need for more comprehensive studies to establish standardized protocols and explore alternative approaches, addressing the risks associated with current treatments and ultimately improving the management of major keloids.

## Supplemental Material

This article contains supplemental material located online at https://doi.org/10.1093/asjof/ojae129.

## Supplementary Material

ojae129_Supplementary_Data
