# Correction to: Wound Coverage, Adjuvant Treatments, and Surgical Outcomes for Major Keloid Scars: A Systematic Review and Meta-Analysis

**DOI:** 10.1093/asjof/ojag038

**Published:** 2026-02-26

This is a correction to: David Cardenas, Trey Cinclair, Anca Dogaroiu, Chandler Hinson, Matthew Sink, Brandon Bruce, Andrei Odobescu, Douglas Sammer, Andrew Y Zhang, Wound Coverage, Adjuvant Treatments, and Surgical Outcomes for Major Keloid Scars: A Systematic Review and Meta-Analysis, *Aesthetic Surgery Journal Open Forum*, Volume 7, 2025, ojae129, https://doi.org/10.1093/asjof/ojae129

The last name of the second co-author is corrected to read: “Trey Cinclair”.

