# Supplementary material for: Wound Coverage, Adjuvant Treatments, and Surgical Outcomes for Major Keloid Scars: A Systematic Review and Meta-Analysis
Source: Aesthet Surg J Open Forum. 2024 Dec 26;7:ojae129. doi: 10.1093/asjof/ojae129 (PMC11811037; doi:10.1093/asjof/ojae129)
Supplement: ojae129_Supplementary_Data [file ojae129_supplementary_data.docx]

**Appendix 1**: Search string

1. **Medline**

Keloid OR (keloid* or cheloid*). Ti,ab,kf OR (major keloid* or major cheloid*).ti,ab,kf. OR (large keloid* or large cheloid*).ti,ab,kf. OR keloid/su [Surgery] OR (surgical* or surgery*).ti,ab,kf.

1. **Embase**

Exp Keloid/ OR (keloid* or cheloid*).ti,ab,kf. OR (major keloid* or major cheloid*).ti,ab,kf. OR (large keloid* or large cheloid*).ti,ab,kf. OR exp Keloid/su [Surgery] OR (surgical* or surgery*).ti,ab,kf.
